# An Automated Inner Dimensional Measurement System Based on a Laser Displacement Sensor for Long-Stepped Pipes

**DOI:** 10.3390/s120505824

**Published:** 2012-05-04

**Authors:** Fumin Zhang, Xinghua Qu, Jianfei Ouyang

**Affiliations:** State Key Laboratory of Precision Measuring Technology and Instruments, Tianjin University, Tianjin 300072, China; E-Mails: quxinghua@tju.edu.cn (X.Q.); oyj@tju.edu.cn (J.O.)

**Keywords:** laser displacement sensor, pipe interior geometry, automated measurement, long-stepped pipe

## Abstract

A novel measurement prototype based on a mobile vehicle that carries a laser scanning sensor is proposed. The prototype is intended for the automated measurement of the interior 3D geometry of large-diameter long-stepped pipes. The laser displacement sensor, which has a small measurement range, is mounted on an extended arm of known length. It is scanned to improve the measurement accuracy for large-sized pipes. A fixing mechanism based on two sections is designed to ensure that the stepped pipe is concentric with the axis of rotation of the system. Data are acquired in a cylindrical coordinate system and fitted in a circle to determine diameter. Systematic errors covering arm length, tilt, and offset errors are analyzed and calibrated. The proposed system is applied to sample parts and the results are discussed to verify its effectiveness. This technique measures a diameter of 600 mm with an uncertainty of 0.02 mm at a 95% confidence probability. A repeatability test is performed to examine precision, which is 1.1 μm. A laser tracker is used to verify the measurement accuracy of the system, which is evaluated as 9 μm within a diameter of 600 mm.

## 1. Introduction

Recent years have seen a growing need for an inner measurement system intended for the quality assessment of large-diameter pipes used in a number of industries, such as nuclear power plants and shipping [1–3]. Shaft and pipe assembly significantly influences the quality of ships. Usually, the stern tube used in ships is circular, with an inner diameter of 0.6 m to 1 m, and a diameter tolerance of 0.03 mm to 0.05 mm [4]. Although pipe sizes vary considerably, most of these pipes are too long and narrow for direct human access. Therefore, conventional measurement tools such as coordinate measuring machines (CMMs), laser trackers, photogrammeters, or inner-diameter micrometers are inappropriate in the field because these all require human intervention. In-pipe automated measurement equipment robots are proven suitable alternatives to current pipe inspection techniques; they are reliable over long sections of pipes and provide sufficient location information [5]. Automated pipe inspection can reduce the manpower required in this process. In-pipe robot prototypes based on different motion mechanisms have been proposed. The prototypes include wheeled, snaking, walking, worming, and helical-drive types. These robots are used for maintaining and repairing pipes, measuring positions of manholes, detecting the location and extent of damage in sewage systems, and measuring the deformations of pipes.

The general profiling approach is to mount a sensor on a mobile vehicle; the sensor can be propelled along the pipe and record its dimensions [6,7]. A wide range of sensors with specific advantages and disadvantages are available. Such sensors include optical method sensors, mechanical stylus, color cameras, ultrasonic and microwave sensors, infrared thermographs, and ground-penetrating radars [8]. To improve accuracy and obtain high-resolution measurements of pipe profiles, laser displacement-sensor rotating techniques based on optical triangulation can be used [9,10]. However, two crucial problems are encountered in stern tubes:

1. Pipes with stepped holes cause tilting of the measurement system, and then the rotation plane of the sensor becomes non-perpendicular to the pipe axis. Data on an ellipse are acquired, making data processing more complex.
2. Pipe diameters vary considerably, so measuring surface data with high accuracy for every pipe specification is difficult to accomplish.

To overcome these problems, we propose a novel automated pipe measurement method and prototype system. The method and system are anticipated to be particularly beneficial to the high-precision measurement of various large-diameter stepped pipes.

## 2. Principle and Components of the Measurement System

**Figure 1.**
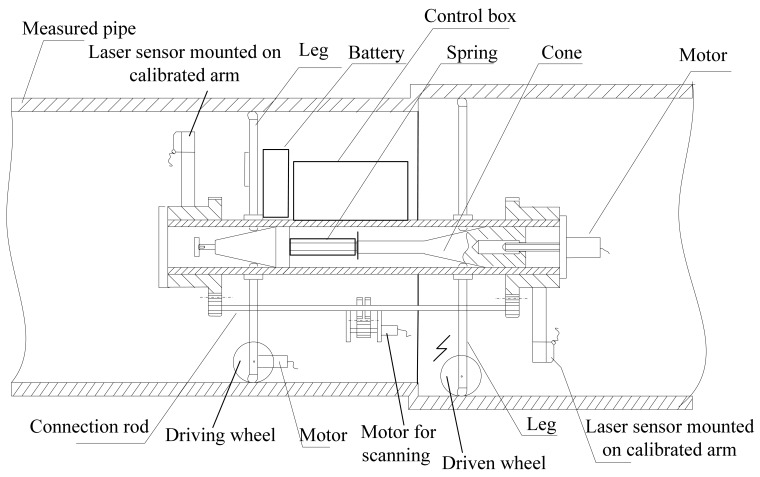
Mechanical structure of the proposed measurement system.

Measurement devices based on mobile wheeled platforms equipped with optical triangulation sensors are constructed to measure the dimensions of the entire interior of a pipe. The measurement is conducted via fast 3D surface scanning. The hardware of a measurement system is composed primarily of four sub-systems, shown in Figure 1 along with the structure of the proposed measurement system.

### 2.1. Scan Units

The measurement principle of a laser displacement sensor, which uses a semiconductor laser and CCD, is triangulation. Optical triangulation displacement sensors have been widely used in industrial measurement applications because they perform non-contact and fast scan measurements with sub-micrometre resolution. However, they have some disadvantages such as shadowing area. The CCD cannot detect points on the surface around the steps, due to the large angular separation of the projection and detection axes. Fortunately, continuous profile measurements are unnecessary. In general, data points on two circle-sections are adequate for obtaining the dimensions of a pipe with the same diameter. Another disadvantage is that the sensors are sensitive to surface characteristics such as material, reflectivity and roughness. However the sensors have good repeatability for specific tasks, and systematic errors introduced by surfaces can be corrected in advance. Therefore optical triangulation displacement sensors are appropriate for this measuring system. The choice of displacement sensor type involves a trade-off between accuracy and measurement range. Therefore, the relative measurement principle based on a laser sensor mounted at the end of an extended arm with appropriate fixtures is used in the proposed system to measure large diameters with high accuracy. The sensor selected in this study is a Keyence LK-G30, which has a measurement distance of 30 mm, resolution of 0.05 μm, and measurement limit of ±5 mm for better linearity (±0.05%). Therefore, the confidence interval of the laser sensor is 0.0025 mm giving about 95% confidence probability. Hence the standard uncertainty component *u*_1_ = 0.0025/2 = 0.0013 mm.

Given that the ability to measure in every radial direction is essential for quantifying the inner wall dimension, a 360° scanner is designed to acquire point data on the part. The extended arm is circumferentially rotated by a servo motor with a 2,048 line/round encoder and high performance bearing. The measurement system can be expressed in cylindrical coordinates (*R, θ, Z*), where *Z* is a linear coordinate of the longitudinal axis of the pipe. In a coordinate system *XOY*, origin *O* is placed at the center of rotation. Rotation angle *θ* from its reset position is measured with an optical encoder, so that the polar coordinates of the measured point in each section can be determined. The mechanism has a range of 360° around the axis. Radius *R*(*θ*) from the pipe center to wall corresponding angle *θ* is:

(1) $$R(\theta )=r_{0}+r(\theta )$$

where *r*(*θ*) is the sensor reading and *r*_0_ is the length of the extended arm. The length *r*_0_ can be varied by changing arm with different length to adapt to different inner diameter of pipes. For example, we choose *r*_0_ = 970 mm in the application of the stern tube with an inner diameter of 1 m. The points measured from the polar coordinate system are converted to the Cartesian coordinate system, and the coordinate of the corresponding point is determined as follows:

(2) $$\left\{ \begin{array}{l} x(\theta_{i})=R(\theta_{i})\cos\theta_{i}\\ y(\theta_{i})=R(\theta_{i})\sin\theta_{i} \end{array}\right.$$

The rotation speed of the motor can be determined in advance by surface characteristics. The measurement time of the sensor in each point is set to 20 μs to 1,000 μs. Diameter and roundness parameters are then calculated using data processing algorithms based on the least-squares fitting algorithm.

The system simultaneously provides either or both section data on the pipe interior. The synchronous rotation of the two symmetric sensors is achieved via a connection rod, thereby increasing efficiency and symmetry.

The rotation accuracy depends not only on a rotary encoder, but also on the dynamic errors of mechanical structures during the rotation of the laser sensor, including inevitable radial runout errors of axes. The displacement introduced by the angle measurement is determined by a micrometer and minimized to approximately 0.01 mm by iterated measurements and sensor rotations. This effect is estimated as a rectangular distribution, hence the standard uncertainty component:

$$u_{2}=0.01/3^{1/2}=0.0058\text{mm}$$

### 2.2. Axial Forward-Backward Walk Units

To satisfy the requirement for forward/backward maneuvering along the pipeline axis to target more sections in a long pipe, we design the measurement system as a four-wheeled carrier, which includes two driving wheels and two driven wheels. Each driving wheel is driven by a motor with an encoder and reducer. A type of MAXON motor, whose torque of 7 Nm capable of providing sufficient force for smooth propulsion on the inner walls of a pipe, is used. Wheels with deep grooves are employed to prevent slipping during system motion. The motion speed of the system can be defined through the motor speed. The accurate position of the measurement section of the system in the pipe is obtained using encoders on the vehicle axes. The orientation of the system is adjusted by independently controlling the driving wheels of the two sides.

### 2.3. Position Fixing and Centering Units

The device has to be fixed with respect to the pipe wall during sensor rotation and pipe sampling for stable axis rotation. The position-fixing mechanism is composed of two sections, each with three legs circumferentially placed 120° apart symmetrically around the longitudinal axis. A pantograph mechanism in six legs is employed to ensure lifting or lowering of the system axis. A motor drives the cone forward, which in the central shaft can radially push six legs until contact with the inner wall of the pipe, and the motor reverses the cone back to six legs once the scanning procedure is complete.

A critical factor in the system is ensuring that the central axis of rotation of the laser sensors is coaxial with the central longitudinal axis of the pipe. Thus, the pantograph design also aids the displacement direction of two sensors perpendicular to the pipe axis during sensor rotation; this feature avoids the acquisition of elliptical data that results in complex data processing. In particular, if a conventional fixing mechanism such as an electromagnet is used, the stepped hole results in the slight deviation of the system axis from the pipe axis. The spring mechanism enables the legs in every section to expand to different lengths to guarantee that the two sections are centered in various diameter holes. Data are directly acquired in the polar coordinate system. Coincidence of the two axes makes the captured data vary within a small working range of the sensor, thereby benefitting error uniformity in the sensor in all radial directions. The error of centering is subject to variations within the range of ±0.01 mm. Therefore, the uncertainty component is estimated as a rectangular distribution:

$$u_{2}=0.01/3^{1/2}=0.0058\text{mm}$$

The drive current of the motor is beneficial for indirectly controlling the stable pressure of the wheels against the pipe wall to obtain a stable central axis. Furthermore, the drive current can also prevent the mechanism from overloading.

### 2.4. Central Control Units

A remotely controlled system is mounted. The control design for this instrument entails a number of different elements. The displacement signal produced by the laser sensor is sent to a PC after analog-to-digital conversion. A controller area network (CAN) field bus is used to control the above-mentioned four motor drivers, including a scanning motor, a centering motor, and two driving wheel motors. The measurement system can remove a cable, which comprises communication, sensor signal, and power supply lines between the mobile robot and off-board computer. Laser sensor and motor encoder data are transmitted to the PC via the Zigbee wireless data communication technique. Freedom from a power supply cable is achieved using a battery as the power supply for the driving motors and the laser sensors. The measured 3D data can be stored in a database and displayed on the graphic user interface.

## 3. Systematic Error Source Analysis and Uncertainty Evaluation

### 3.1. Systematic Error of Arm Length

Extended arms with corresponding lengths are assembled for various diameters. The length of extended arm *r*_0_ is calibrated in advance by a caliper. The uncertainty associated with arm calibration is *u_L_*_0_ = 0.01 mm (2σ). However, the effect of environmental disturbance dramatically diminishes measurement precision. Therefore, to maintain length stability between calibration and ultimate use, this systematic error is corrected using Equation (3):

(3) $$L=L_{0}(1+\alpha \Delta T)$$

The uncertainty associated with the calibrated absolute arm length *L* is:

(4) $$u_{L}=\sqrt{{\left(1+\alpha \Delta T\right)}^{2}u_{L0}^{2}+{\left(L_{0}\Delta T\right)}^{2}u_{\alpha }^{2}+{\left(L_{0}\alpha \right)}^{2}u_{T}^{2}}$$

For example, *L*_0_ = 500 mm. The invar arm has a very low coefficient of thermal expansion α = 1 × 10^−6^/°C, with an uncertainty of *u*_α_ = 5%α (2σ). The uncertainty associated with the temperature measurement is *u_T_* = 0.5 °C (2σ). When the temperature difference is Δ*T* = 20 °C, the arm length is corrected by *L* = *L*_0_ (1 + αΔ*T*) = 500.01 mm, with an uncertainty of *u_L_* = 0.01 mm (2σ). So standard uncertainty is *u*_4_ = 0.005 mm.

Temperature influences not only arm length but also pipe diameter. Fortunately, compensation for this error is unnecessary. The purpose of stern tube measurement is to provide guidance for subsequent shaft manufacture with a suitable diameter. Hence, the temperature sensor in the control box is added to calculate the shaft diameter and consequently enable strict shaft and pipe assembly under the same temperature.

### 3.2. Systematic Error of Tilt and Offset

**Figure 2.**
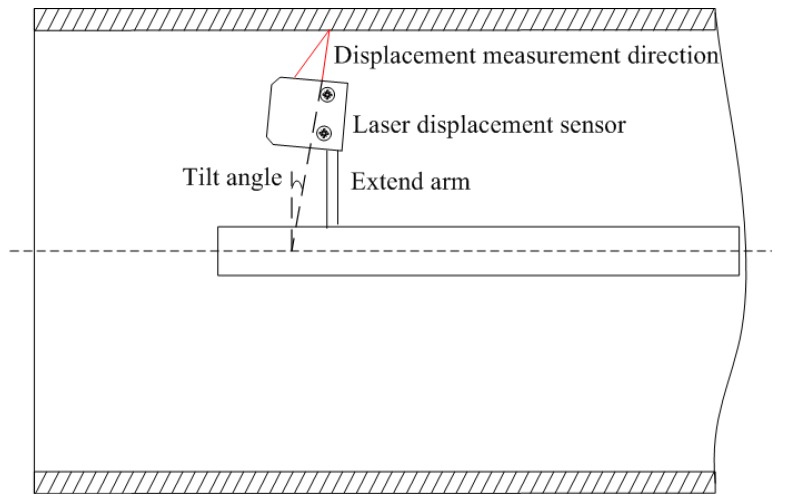
Tilt and offset systematic errors.

The relationship between the incident laser beam displacement measurement and the Z-axis influences the uncertainty associated with the displacement measurement. Initially, the two axes are assumed intersecting and perpendicular. However, tilt and offset systematic errors exist, as shown in Figure 2.

**Figure 3.**
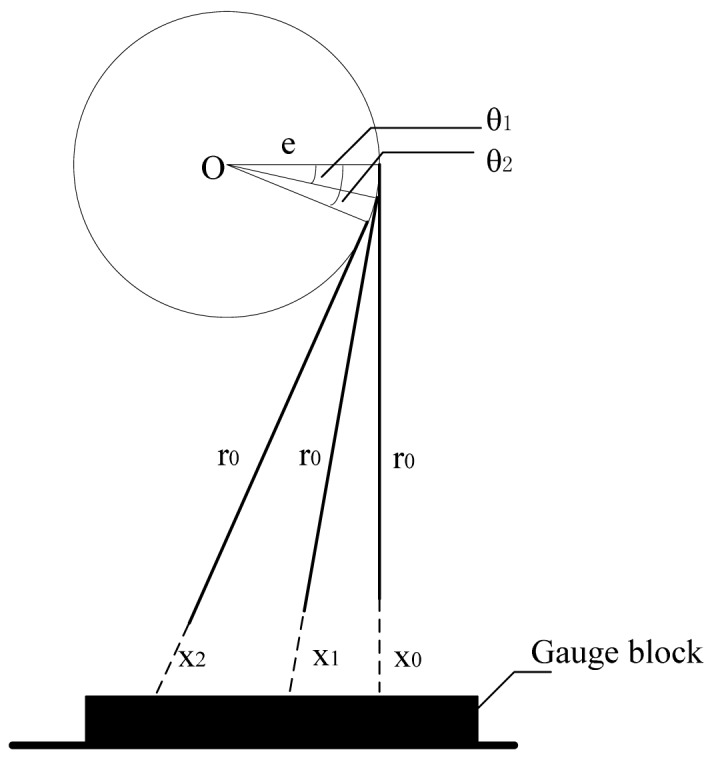
Calibration of offset systematic error.

A calibration procedure is designed (Figure 3). The offset is denoted as e. By measuring a plane of the gauge block, the minimum sensor reading is found as *x*_0_. After rotating *θ*_1_ and *θ*_2_, the sensor reading is *x*_1_ and *x*_2_.

Equation (5) is then obtained:

(5) $$\left\{ \begin{array}{l} r_{0}+x_{0}=H\\ e\sin\theta_{1}+(r_{0}+x_{1})\cos\theta_{1}=H\\ e\sin\theta_{2}+(r_{0}+x_{2})\cos\theta_{2}=H \end{array}\right.$$

The calibration parameter is also derived:

(6) $$\left\{ \begin{array}{l} r_{0}=\frac{x_{1}\cos\theta_{1}\sin\theta_{2}+x_{2}\cos\theta_{2}\sin\theta_{1}+x_{0}(\sin\theta_{2}-\sin\theta_{1})}{\sin\theta_{1}-\sin\theta_{2}-\cos\theta_{2}\sin\theta -\cos\theta_{1}\sin\theta_{2}}\\ e=\frac{r_{0}+x_{0}-(r_{0}+x_{1})\cos\theta_{1}}{\sin\theta_{1}} \end{array}\right.$$

Subsequently, we correct the conversion to Cartesian coordinates Equation (2) for systematic errors:

(7) $$\left\{ \begin{array}{l} x(\theta_{i})=[r_{0}+r(\theta_{i})]\cos[\theta_{i}+\arctan(\frac{e}{r_{0}+r(\theta_{i})})]\\ y(\theta_{i})=[r_{0}+r(\theta_{i})]\sin[\theta_{i}+\arctan(\frac{e}{r_{0}+r(\theta_{i})})] \end{array}\right.$$

Possible correlation of the uncertainty components is weak, so can be neglected. The combined standard uncertainty is estimated by:

$$u_{c}=\sqrt{u_{1}^{2}+u_{2}^{2}+u_{3}^{2}+u_{4}^{2}}=\sqrt{0.0013^{2}+0.0058^{2}+0.0058^{2}+0.005^{2}}=0.01\text{mm}$$

The expanded uncertainty, with about 95% coverage probability, is estimated as *U*_95_ = 2*u*_c_ = 0.02 mm. To summarize, a complete uncertainty budget for the measurement system is given in Table 1.

## 4. Measured Results

**Figure 4.**
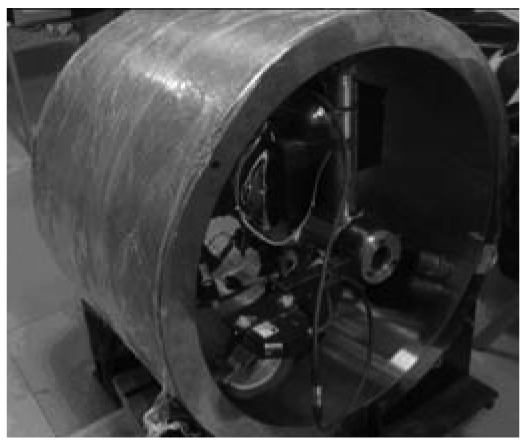
Experiment on the measurement system in the laboratory.

This system is pulled through the pipes to assess its operation and automation performance, precision, and accuracy. The first test (Figure 4) is implemented in horizontal pipelines with diameters of 582 mm in the laboratory. Two profiles are collected.

**Figure 5.**
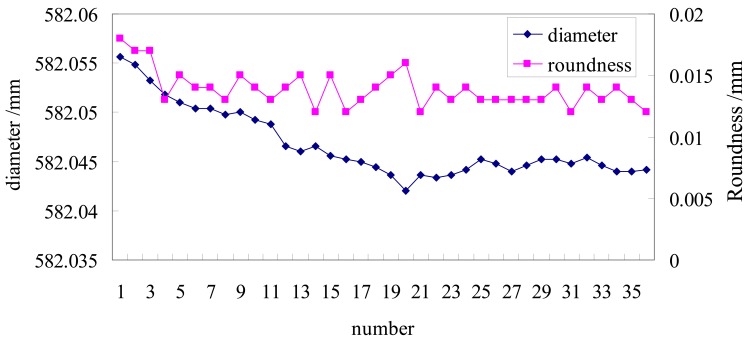
Repeatability of the measurement system.

The test involves measurements continually repeated 36 times, with a sampling interval of 2 min. Figure 5 shows the results.

The repeatability indicator is given by the standard deviation σ = 3.48 μm. Additionally, an obvious warm-up phase can be observed, which lasts approximately 26 min (which corresponds to the warm-up time indicated in the sensor specifications). Mechanical assemblies within the sensors are sensitive to the variation of temperature during the warm-up period, resulting in the drift of the peak or centroid position of the light intensity distribution imaged on the CCD, and then introducing displacement error. For example, the cavity length of semiconductor laser varying with temperature will make the laser wavelength and optical power fluctuation of the light source. Hence enough warm-up time is necessary. If the system is used without waiting for the warm-up to be over, this phenomenon should also be taken into account when assessing the uncertainty. After 26 min, the data has stabilized and the next data points have a standard deviation σ = 1.10 μm. Considering the difficulty in measuring the temperature of the semiconductor cavity in the laser sensor, two methods are used for overcoming drift. First, the trend of the drift is predicted by linear regression of the data to achieve software compensation. Second, in fact, during this phase, drift error are actually variable between 2 μm and 10 μm and quite different from those observable after the instrument has warmed up. Hence warming up the instrument for at least 20 min is the most common and reliable method for laser-based sensors.

**Figure 6.**
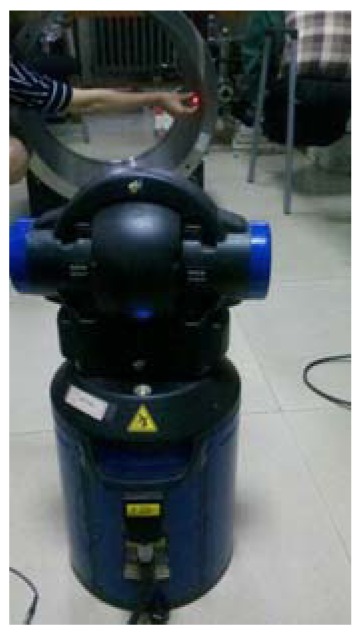
Calibration using a laser tracker.

The differences between the diameters measured 36 times and nominal diameters measured with the tracker are calculated for accuracy evaluation (Figure 6). According to manufacturer's performance specifications of the laser trackers, the distance measurement uncertainty component (2σ) of the laser interference system is 10 μm + 0.8 μm/m × 2 m = 0.0116 mm, while the angular measurement uncertainty component (2σ) is 18 μm + 3 μm/m × 2 m = 0.024 mm. Table 2 shows the average results.

The maximum deviation is 6 μm, which reflects the residual systematic error.

**Figure 7.**
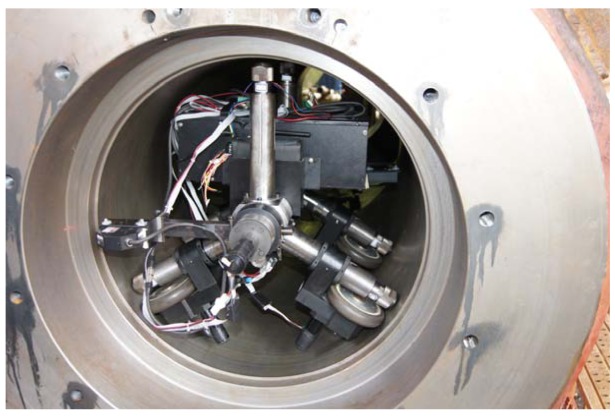
Field experiment on the measurement system.

**Figure 8.**
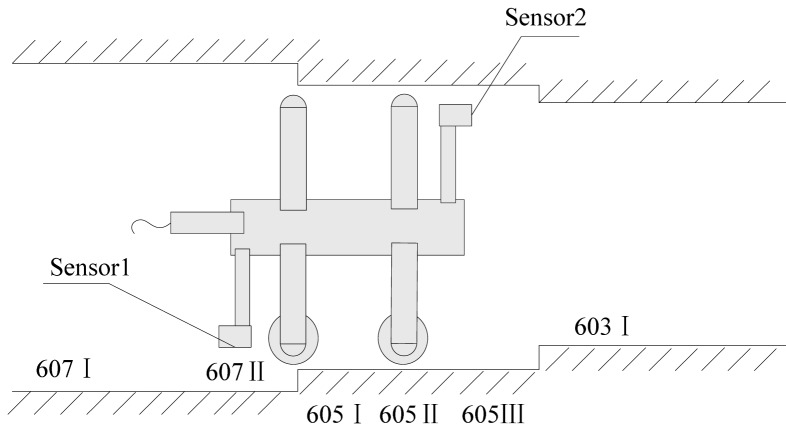
Position of the six profiles.

The second test is implemented in horizontal stepped pipelines with diameters of 605 mm in the field, where a total of six profiles are collected (Figures 7 and 8).

Given the diameters beyond the range of the above-mentioned arms and legs, other arms and legs of different lengths are assembled on site to measure the corresponding diameters. This process results in altered assembly parameters. Consequently, the systematic error requires recalibration. Table 3 lists the average results derived by sensor 2 before and after calibration, compared with those obtained by the laser tracker. The maximum deviation is 9 μm. Because of the sufficient length of the pipe, the same section can be scanned from two sensors. Table 4 shows the comparison of the results of two sensors.

The maximum deviation between the two sensors is 4 μm.

## 5. Conclusions

This paper develops a metrology system for the interior geometry of large-sized pipes. The system facilitates automatic 3D measurements in all radial directions with a cylindrical coordinate measurement method. It consists of a laser beam scanning sensor carried by a motorized carriage, circuits for signal processing and control, as well as a PC for displaying results and setting parameters. A fixing and centering mechanism based on two sections is proposed to avoid elliptical measurements introduced by stepped holes. Measurements can be remotely performed in an automated mode because of the wireless system. The systematic assembly errors, such as arm length, tilt, and offset errors, are calibrated.

The expanded uncertainty, with about 95% coverage probability, is evaluated as 0.02 mm. Its effectiveness is illustrated with experimental results. In laboratory and field tests, a number of cross-sections are measured by this system, and the main specifications of the system are subsequently analyzed. A repeatability test is conducted to examine precision, which is 1.1 μm. This result means that random errors of both laser sensor measurement and rotary runout are small enough, but this experiment does not reflect centering accuracy. Therefore the scan test at the same section from the two sensors is performed, and the maximum deviation is 4 μm. this result suggests the reproducibility that represents centering accuracy besides repeatability, due to the existing of repeat fixing and centering process. A laser tracker is used to test the measurement accuracy of the system, which is evaluated as 9 μm within a diameter of 600 mm. It is also of crucial importance to take into account the warm-up phase of the instruments. The measurement accuracy for large-sized pipes is significantly improved given the addition of an extended arm with absolute length to the laser displacement measurement within a small range. In principle, the measurement range of the system can reach a greater range of more than 1,000 mm with high accuracy by increasing leg and arm length.

## Figures and Tables

**Table 1.** Uncertainty budget for the inner pipe measurement system (mm).

**Component**	**Value**	**Distribution**	**Standard uncertainty**
Sensor	0.0025	Normal	0.0013
Rotation	0.01	Rectangular	0.0058
Centering	0.01	Rectangular	0.0058
Arm length	0.01	Normal	0.005
Combined standard uncertainty	-	Normal	*u*_c_ = 0.01
Expanded uncertainty at 95% confidence level	*U*_95_ = 0.02	Normal, k = 2	-

**Table 2.** Results measured in the laboratory (mm).

**Position**	**Sensor**		**Laser tracker**	

	**Diameter**	**Roundness**	**Diameter**	**Roundness**
I	582.0413	0.013	582.0436	0.0141
II	584.0912	0.016	584.097	0.0142

**Table 3.** Diameters measured in the field before and after calibration (mm).

**Position**	**Before calibration**	**After calibration**	**Laser tracker**
605 I	605.0997	605.0724	605.0794
605 II	605.0988	605.0658	605.0752
605 III	605.0956	605.0622	605.0661
603 I	603.2452	603.2176	603.2261

**Table 4.** Results measured in the field using the two sensors (mm).

**Position**	**Sensor 1**		**Sensor 2**	

	**Diameter**	**Roundness**	**Diameter**	**Roundness**
605 I	605.0685	0.02	605.0724	0.01
605 II	605.0637	0.02	605.0658	0.013
605 III	605.0635	0.014	605.0622	0.014

## References

[1] Bright G., Ferreira D., Mayor R. (1997). Automated pipe inspection robot. Ind. Robot.

[2] Zhu C. (2007). In-pipe robot for inspection and sampling tasks. Ind. Rob.

[3] Zhang G.Y., Xu X.P., Fu X.H. (2002). The measurement and control of diameter in large-scale part processing. J. Mater. Process. Technol.

[4] Ma Z., Hu Y., Huang J. (2007). A novel design of in pipe robot for inner surface inspection of large size pipes. Mech. Based Des. Struct. Mach.

[5] Zhang Y.W., Yan G.Z. (2007). In-pipe inspection robot with active pipe-diameter adaptability and automatic tractive force adjusting. Mech. Mach. Theory.

[6] Duran O., Althoefer K., Seneviratne L.D. (2002). State of the art in sensor technologies for sewer inspection. IEEE Sens. J.

[7] Clarke T.A., Grün A., Kahmen H. (1995). The Development of an Optical Triangulation Pipe Profiling Instrument. Optical 3-D Measurement Techniques III.

[8] Kim K.C., Oh S.B., Kim S.H., Kwak Y.K. (2001). Design of a signal processing algorithm for error-minimized optical triangulation displacement sensors. Meas. Sci. Technol.

[9] Zhang Z.F., Feng Q.B., Gao Z. (2008). A new laser displacement sensor based on triangulation for gauge real-time measurement. Opt. Laser Technol.

[10] Zhang W.W., Zhuang B.H. (1998). Non-contact laser inspection for the inner wall surface of a pipe. Meas. Sci. Technol.

